# Optimal external laryngeal manipulation versus McCoy blade in active position in patients with poor view of glottis on direct laryngoscopy

**DOI:** 10.4103/0019-5049.60497

**Published:** 2010

**Authors:** Arumugam Vasudevan, Ranjani Venkat, Ashok Shankar Badhe

**Affiliations:** Departments of Anesthesiology and Critical Care, Jawaharlal Institute of Postgraduate Medical Education and Research (JIPMER), Puducherry, India

**Keywords:** Difficult intubation, McCoy laryngoscope, OELM, Optimal external laryngeal manipulation

## Abstract

Successful endotracheal intubation requires a clear view of glottis. Optimal external laryngeal manipulation may improve the view of glottis on direct laryngoscopy with Macintosh blade, but it requires another trained hand. Alternatively, McCoy laryngoscope with elevated tip may be useful. This study has been designed to compare the two techniques in patients with poor view of glottis. Two hundred patients with ‘Grade 2 or more’ view of glottis on direct laryngoscopy with Macintosh blade are included in the study. Optimal external laryngeal manipulation was applied, followed by laryngoscopy with McCoy blade in activated position; and the view was noted in both situations. The two interventions were compared using Chi-square test. The overall changes, in the views, were analyzed with Wilcoxon signed rank test. Both the techniques improved the view of glottis significantly (*P*<0.05). Optimal external laryngeal manipulation was significantly better than McCoy laryngoscope in active position, especially in patients with Grade 3 or 4 baseline view, poor oropharyngeal class, decreased head extension and decreased submandibular space (odds ratio = 2.36, 3.17, 3.22 and 26.48 respectively). To conclude, optimal external laryngeal manipulation is a better technique than McCoy laryngoscope in patients with poor view of glottis on direct laryngoscopy with Macintosh blade.

## INTRODUCTION

Successful endotracheal intubation requires a clear view of glottis. The Macintosh laryngoscope is the most commonly used device for direct laryngoscopy. When the view of glottis is poor, manoeuvres like optimum external laryngeal manipulation (OELM) applied either by an assistant or with the laryngoscopist's right hand, may improve the view.[1] McCoy and Mirakhur reported a modification of the Macintosh blade, popularly known as the McCoy blade, which could aide in improving the view of glottis during direct laryngoscopy.[2] Previous reports have shown that McCoy blade[3–8] as well as OELM[1,9] are effective in cases of difficult intubation. This study was undertaken to find out whether one of these techniques is better than the other, in patients with poor view of glottis, and to identify specific airway parameters that may influence the success of either technique.

## METHODS

The study was conducted with institutional approval and informed consent of each patient. The ‘Recommendations guiding medical doctors in biomedical research involving human subjects’ contained in the eighteenth ‘Declaration of Helsinki’ adopted by the World Medical Assembly, Helsinki, Finland, 1964, were adhered to throughout the study. Patients above 15 years of age in ASA Class I and II undergoing elective surgical procedure under general anaesthesia with endotracheal intubation were included in the study.

The Mallampati oropharyngeal Class (Class 1 to 4), interincisor gap, range of head extension (normal or restricted) and mentohyoid distance were noted during preanaesthetic evaluation. Patients were premedicated with Diazepam 0.15 mg/kg, Famotidine 20 mg *p.o.* and Glycopyrrolate 0.2 mg *i.m.* half hour prior to induction. Fentanyl 2 *μ*g/kg was administered three minutes before induction. Head was placed in standard intubating position and anaesthesia induced with Thiopentone 5 mg/kg; maintained with Isoflurane 1% in N_2_O:O_2_ mixture (2:1); neuromuscular blockade achieved with Vecuronium 0.15 mg/kg and lungs were ventilated with bag and mask. After four minutes, direct laryngoscopy was performed using Macintosh blade by an anaesthesiologist blinded to preoperative airway assessment. The view of glottis was noted (Mac-baseline) as per the classification by Cormack and Lehane.[10] In patients with Grade 1 view of glottis tracheal intubation was completed with no further manipulation of the airway and they were excluded from the study.

If the view of glottis was Grade 2 or more, optimal external laryngeal manipulation was applied by a second anaesthesiologist and the best view of glottis achieved noted (Mac-OELM). Lungs were ventilated with mask for 30 seconds and laryngoscopy was performed with McCoy laryngoscope. The view of glottis after levering the tip of the blade was noted (McCoy-active). The change in view of glottis produced by each technique was analyzed with Wilcoxon signed rank test. Comparison between the techniques was done by Chi-square test; *P*<0.05 was considered significant.

## RESULTS

A total of 382 patients were assessed, 182 were excluded because of Grade 1 view; 200 patients were finally included in the study. The patients were aged 48±13 years (mean±standard deviation) (range 17 to 86 years) and weighed 53±8 kg; 75 patients were males and 125 females. The details of preoperative airway assessment are given in Table 1.

**Table 1:** Details of preoperative airway assessment

Parameter		Frequency	Percentage
Mallampati class	1 and 2	94	47
	3 and 4	106	53
Inter incisor gap	≥3.5 cm	189	94.5
	<3.5 cm	11	5.5
Head extension	Normal	162	81
	Restricted	38	19
Mentohyoid distance	>4.5 cm	136	68
	<4.5 cm	64	32

The view of glottis with Macintosh blade (Mac-baseline), optimal external laryngeal manipulation (Mac-OELM) and McCoy blade after levering (McCoy-active) are given in Table 2.

**Table 2:** Frequency table of the ‘view of glottis’ with Macintosh (Mac-baseline), optimal external laryngeal manipulation (Mac-OELM) and McCoy blade in active position (McCoy-active)

View of glottis	Mac-baseline	Mac-OELM	McCoy-active
Grade 1	-	100	73
Grade 2	112	80	84
Grade 3	87	20	37
Grade 4	1	0	6
Total	200	200	200

The details of improvement or worsening of individual grades of view of glottis with either technique as per Wilcoxon signed rank test are given in Table 3. A decrease in the grade is taken as a ‘positive rank’; an increase as a ‘negative rank’ and no change is a ‘tie’. A significant number of patients had improved view of glottis by one or more grade with ‘Mac-OELM’ (n=165, *P*=0.01) as well as with ‘McCoy-active’ (n=105, *P*=0.01). No patient had their view worsened with ‘Mac-OELM’ whereas 12 patients with ‘McCoy-active’ had worse view than ‘Mac-baseline’.

**Table 3:** Details of change in view of glottis with Mac-OELM and McCoy-active from Mac-baseline view

Technique	Change in glottic view			*P*-value
		
	Improved (positive)	Same (tie)	Worsened (negative)	
OELM (vs Macintosh)	165	35	0	0.01
McCoy (vs Macintosh)	105	83	12	0.01

The view of glottis can be classified into easy view (Grade 1 or 2) and difficult view (Grade 3 or 4) for all practical purposes. The number of patients with easy view was significantly (*P*=0.01) higher with ‘Mac-OELM’ than with ‘McCoy-active’ (n=180 and 157 respectively); with an odd ratio of 2.46. This means that the odds of having an easy view are 2.46 to 1 with ‘Mac-OELM’ against ‘McCoy-active’ [Table 4].

**Table 4:** Chi-square table showing comparison between Mac-OELM and McCoy-active in all patients

Category	View of glottis		Chi-square	
		
	Difficult (3 and 4)	Easy (1 and 2)	*P*-value	Odds ratio
McCoy-active	43	157	0.01	2.46
Mac-OELM	20	180		

A total of 88 patients had difficult view with ‘Mac-baseline’. Significantly more number of patients in this subgroup improved from difficult view to easy view with ‘Mac-OELM’ than with ‘McCoy-active’ (n=68 and 52 respectively, *P*=0.01, odds ratio=2.36) [Table 5].

**Table 5:** Chi-square table comparing the two techniques in the subgroup of patients with ‘difficult view’ on baseline laryngoscopy with Macintosh blade

Category	View of glottis		Chi-square	
	Difficult (3 and 4)	Easy (1 and 2)	*P*-value	Odds ratio
McCoy-active	36	52	0.01	2.36
Mac-OELM	20	68		

We compared both the techniques, in each subset of patients, with adverse airway parameters, to identify their influence. The details of change in view of glottis in patients with poor oropharyngeal class (Mallampati Class 3 and 4), restricted head extension, decreased submandibular space (mentohyoid distance <4.5 cm) and decreased mouth opening (Interincisor gap <3.5 cm) are given in Table 6.

**Table 6:** Details of view of glottis with Mac-OELM and McCoy-active in patients with adverse airway parameters

Airway parameter	No. of patients	Improvement		Same/worse		Chi-square	
		
		OELM	McCoy	OELM	McCoy	*P*-value	Odds ratio
Mallampati - Class 3 and 4	106	82	55	24	51	0.001	3.17
Head extension-restricted	38	31	22	7	16	0.02	3.22
Mentohyoid <4.5 cm	64	52	9	12	55	0.01	26.48
Interincisor gap <3.5 cm	11	5	4	6	7	0.66	-

In this study, 106 patients had Mallampati Class 3 or 4; Mac-OELM and McCoy-active could improve the view by at least one grade in 82 and 25 of these patients respectively (*P*=0.001, odds ratio=3.17). Similarly, out of 38 patients with ‘restricted head extension’, improvement was seen in 31 with Mac-OELM and in seven with McCoy-active (*P*=0.02, odds ratio=3.22). Mentohyoid distance was <4.5 cm in 64 patients; while Mac-OELM improved the view in 52 of these patients, McCoy-active could improve only in 12 patients (*P*=0.01, odds ratio=26.48). There was no significant difference between the two techniques in patients with decreased mouth opening.

## DISCUSSION

Mac-OELM as well as McCoy-active resulted in a significant improvement in individual grades of view of glottis over those obtained with the Macintosh blade, but the quantum of improvement was higher with Mac-OELM than with McCoy-active and the number of *difficult views* was less with Mac-OELM when compared to McCoy-active. This was consistent with the finding of an earlier study by Harioka *et al.*, in which the addition of external laryngeal pressure to the Macintosh laryngoscope made the distribution of the laryngoscopic view better than that obtained by the McCoy laryngoscope with the tip in the elevated position.[9]

One patient in our study had Grade 4 view of glottis, which improved to Grade 2 by Mac-OELM, whereas McCoy-active had failed to improve the view. In another study, Randell *et al.* noticed that in 95/100 subjects ‘external laryngeal manipulation’ was better than McCoy blade in a difficult intubation setting[11] Chisholm and Calder had found no improvement 8/9 cases with a Grade 4 laryngeal view when McCoy blade was used in active position.[12]

Levitan and Ochroch, in a videographic examination, had suggested that OELM displaced the larynx backwards, which improved the alignment of the laryngeal axis with the line of sight.[13] Secondly, it helped correctly position the tip of the curved blade in the vallecula. This could then allow for proper pressure on the hyoepiglottic ligament and effective indirect control of the epiglottis. However, external laryngeal pressure required the assistance of another person trained in the application of OELM.

The view of glottis worsened in 12 patients with McCoy-active; six patients had Grade 4 view of glottis with McCoy-active while only one patient had Grade 4 with Mac-baseline and none with Mac-OELM. Chisholm and Calder had found that 5/39 cases with Grade 3 view worsened to Grade 4 with McCoy in active position.[12] Cook and Tuckey had found a deterioration of 43% on grades previously Grade 1 and 2 with the Macintosh blade.[14] The worsening of view could be because of the excessive anterior lifting of the vallecula resulting in movement of the larynx out of the line of vision or because of the movement of body of blade into the line of vision.[15–17] Since, we excluded patients with Grade 1 view of glottis, the total worsening could not be assessed and this probably explained the lower incidence in our study.

Another reason for worsening of view of glottis with McCoy-active could be smaller submandibular space, resulting in a ‘pear drop’ phenomenon during attempts to lift the tongue out of vision.[18–20] Five of the six patients who had Grade 4 view with McCoy-active had decreased mentohyoid distance. The odds of producing an improvement in view of glottis, in patients with decreased submandibular space with McCoy-active was much lower than that with Mac-OELM. Earlier reports have suggested that McCoy laryngoscope was not effective in patients with anterior complex problems.[21]

Mac-OELM improved the view of glottis in more patients than McCoy-active who had restricted head extension or poor oropharyngeal class, whereas McCoy-active was as effective as Mac-OELM in patients with decreased mouth opening. The limitations of our study include - (i) use of a grading system that has inter observer variability and is not an objective method of validating glottic exposure, (ii) the sequence of laryngoscopy with Macintosh, OELM and McCoy was not varied which might have affected the results), (iii) the laryngoscopist could not be blinded to the use of each technique.

## CONCLUSION

OELM with Macintosh blade is a better technique than McCoy blade, with the lever activated in patients having poor view of glottis on direct laryngoscopy using Macintosh blade. McCoy blade in its active position can be considered as a second line device when additional trained personnel are not available. McCoy blade is a poor choice for patients with decreased submandibular space.
